# Liver cirrhosis with encapsulating peritoneal sclerosis after 4 years of peritoneal dialysis: A case report

**DOI:** 10.1097/MD.0000000000028350

**Published:** 2021-12-23

**Authors:** Kanako Watanabe-Kusunoki, Yoshihiro Kusunoki, Junichi Goto, Kazutaka Kukita

**Affiliations:** a Department of Internal Medicine, Kushiro Red Cross Hospital, Kushiro, Japan; b Department of Surgery, Sapporo Hokuyu Hospital, Sapporo, Japan.

**Keywords:** encapsulating peritoneal sclerosis, end-stage renal disease, liver cirrhosis, peritoneal dialysis, surgery

## Abstract

**Rationale:**

Encapsulating peritoneal sclerosis (EPS), or abdominal cocoon, is a rare but fatal syndrome characterized by intestinal obstruction owing to adhesions in a diffusely thickened peritoneum. Long-term peritoneal dialysis (PD) for more than 5 years is commonly associated with EPS, while liver cirrhosis also carries a risk of EPS. However, there have been only a few reports that describe a case of EPS complicated with both cirrhosis and PD. We herein describe a case of advanced liver cirrhosis with end-stage renal disease (ESRD) who developed EPS after 4 years of PD and who was successfully recovered by surgery.

**Patient concerns:**

A 58-year-old man with alcoholic liver cirrhosis suffered abdominal pain. The patient had a 4-year history of continuous cycling PD to manage ESRD as well as cirrhotic complications of refractory ascites and hypotension. Laboratory test results showed increased levels of inflammation, and contrast-enhanced computed tomography scan showed dilated loops of small bowel proximal to the site of intestinal obstruction. The patient was suspected to have developed intestinal obstruction owing to EPS. The patient discontinued continuous cycling peritoneal dialysis and switched to hemodiafiltration.

**Diagnoses:**

Laparoscopy revealed a whitish membranous material wrapped around the bowel, especially at the terminal ileum with a narrowed portion, consistent with EPS.

**Interventions:**

Repeated decortication of fibrous peritoneal membranes successfully released the intestinal obstruction.

**Outcomes:**

The postoperative course went well and abdominal pain remained in remission. Because abdominal distension owing to ascites got intolerable in a few days after surgery, a PD catheter was re-inserted and ascitic fluid drainage was resumed with peritoneal lavage. The patient continued hemodiafiltration using vasopressor agents.

**Lessons:**

The Cirrhotic patient with ESRD undergoing PD could develop EPS after a short duration of PD.

## 1 Introduction

Encapsulating peritoneal sclerosis (EPS) is a rare but potentially life-threatening syndrome with a high mortality rate of approximately 40%.^[1]^ EPS is characterized by intestinal obstruction owing to adhesions in a diffusely thickened peritoneum.^[2]^ Long-term peritoneal dialysis (PD) is commonly associated with EPS; almost always occurs in patients treated for more than 5 years.^[3]^ While a variety of other causes including liver cirrhosis have been reported.^[4,5]^ In advanced liver cirrhosis, renal vasoconstriction leads to hepatorenal syndrome followed by end-stage renal disease (ESRD), and 5% to 6% of patients with ESRD have cirrhosis when initiating renal replacement therapy.^[6]^ Although hemodialysis is the predominant dialysis modality in cirrhotic patients with ESRD, PD has recently been recognized as a feasible therapeutic option for those patients to avoid hemodialytic complications such as unstable hemodynamics, as well as to control refractory ascites.^[7,8]^

Long-term PD and cirrhosis carry a risk of EPS respectively, and cirrhosis is associated with ESRD, however, there have been only a few reports that describe EPS complicated with both cirrhosis and PD. Here we present a case of advanced liver cirrhosis with ESRD who developed EPS after 4 years of PD and who was successfully recovered by surgery.

## 2 Case presentation

A 58-year-old Japanese man was diagnosed with alcoholic cirrhosis with esophageal varices 9 years previously. Because the refractory ascites gradually worsened, and chronic kidney disease owing to hepatorenal syndrome developed to ESRD, he began continuous cycling PD (CCPD) 4 years previously to manage the renal disease as well as the cirrhosis complications of ascites and hypotension. He experienced peritonitis five times during 4 years of CCPD. Because he experienced recurrent hepatic encephalopathy under medications and CCPD with an additional exchange of dialytic fluid, combined therapy with CCPD and hemodiafiltration (5 days of CCPD combined with one hemodiafiltration per week) was initiated 1 month prior to presentation, which resulted in less frequent encephalopathy episodes with lower ammonia concentrations. At the same time, because the patient's hepatic reserve declined and reached Child–Pugh class C, he was registered for brain death hepatorenal transplantation.

**Figure 1:**
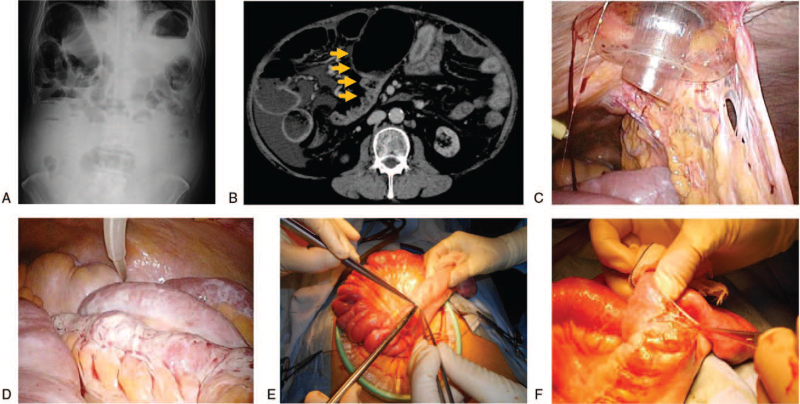
Imaging studies and surgical findings. (A) Plain abdominal radiography showing dilated bowel loops with air-fluid levels. (B) Contrast-enhanced computed tomography scan showing dilated loops of small bowel proximal to the site of obstruction near the ileocecum (arrowheads), with no peritoneal calcification or thickening. (C) The peritoneal dialysis catheter is surrounded by intraperitoneal adhesions. (D) Laparoscopy showing a whitish membranous material wrapped around the bowel. (E and F) A stricture of the terminal ileum is visible, covered with sclerotic peritoneum, which was carefully decorticated.

**Figure 2:**
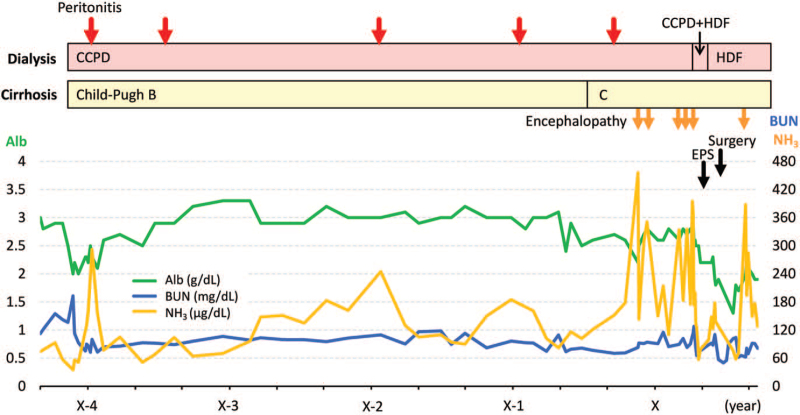
The patient's clinical course. Alb = albumin, BUN = blood urea nitrogen, EPS = encapsulating peritoneal sclerosis, HDF = hemodiafiltration, NH_3_ = ammonia, CCPD = continuous cycling peritoneal dialysis.

The patient suffered abdominal pain and was admitted to our department. Plain abdominal radiography revealed dilated bowel loops with air-fluid levels (Fig. 1A). Three days after bowel rest and discontinuing CCPD, the abdominal pain improved. He restarted dietary and combined dialysis therapy; however, his abdominal pain relapsed in 2 weeks. Laboratory test results (Table 1) showed elevated C-reactive protein concentrations, and contrast-enhanced computed tomography scan revealed dilated loops of small bowel proximal to the site of obstruction near the ileocecum (Fig. 1B). According to the physical examination, laboratory, and image study findings, the patient was suspected to have developed intestinal obstruction owing to EPS. The recurrent peritonitis episodes and the results for the high peritoneal solute transport rate (the dialysate to plasma concentration ratio for creatinine at 4 hours was 0.896) supported the suspicion of EPS. He discontinued CCPD and was transferred for surgery. Laparoscopy revealed a PD catheter surrounded by intraperitoneal adhesions (Fig. 1C), which was removed. A whitish membranous material was shown to wrap around the bowel (Fig. 1D), especially at the terminal ileum with a narrowed portion (Fig. 1E), consistent with EPS. The sclerotic peritoneum was carefully decorticated (Fig. 1F), resulting in the release of the bowel from peritoneal adhesions. The postoperative course went well; however, he returned to our hospital when abdominal distension owing to ascites worsened a few days after discontinuing the intraperitoneal drainage. Even though ascitic fluid drainage at catheter re-placement could lead to peritonitis or unstable hemodynamics owing to albumin leakage, the patient wished to alleviate his abdominal symptoms by frequent ascitic fluid drainage using a catheter. Therefore, a PD catheter was re-inserted, and ascitic fluid drainage was resumed with peritoneal lavage. He was discharged on postoperative day 38. His symptoms of abdominal pain and distension remained in remission, and he continued hemodiafiltration using vasopressor agents during the 3-month follow-up (Fig. 2).

**Table 1:** Laboratory data.

Complete Blood Count		
WBC	4990	/μL
RBC	2.91	×10^6^/μL
Hb	10.2	g/dL
Plt	7.6	×10^4^/μL
Biochemistry		
TP	6.3	g/dL
Alb	2.3	g/dL
T-Bil	0.58	mg/dL
AST	16	U/L
ALT	10	U/L
LDH	232	U/L
ALP	829	U/L
γ-GTP	21	U/L
CK	37	U/L
BUN	75.2	mg/dL
Cr	8.4	mg/dL
UA	5.7	mg/dL
Na	133	mEq/L
K	3.3	mEq/L
Cl	98	mEq/L
Ca	8	mg/dL
P	5.6	mg/dL
NH_3_	137	μg/dL
CRP	3.05	mg/dL
Coagulation		
PT	85	%

ALP = alkaline phosphatase, Alb = albumin, ALT = alanine aminotransferase, AST = aspartate aminotransferase, BUN = blood urea nitrogen, CK = creatine kinase, Cr = creatinine, CRP = c-reactive protein, γ-GTP = gamma-glutamyltransferase, Hb = hemoglobin, LDH = lactate dehydrogenase, NH3 = ammonia, Plt = platelet, PT = prothrombin time, RBC = red blood cell, T-Bil = total bilirubin, TP = total protein, UA = uric acid, WBC = white blood cell.

## 3 Discussion

The present case of liver cirrhosis with ESRD developed EPS after 4 years of PD. Surgical therapy successfully released the intestinal obstruction and rescued the patient. PD was a favorable dialysis modality for the patient to manage renal failure as well as cirrhotic complications of refractory ascites and unstable hemodynamics, however, the patient needed to discontinue PD after EPS and continued hemodiafiltration using vasopressor agents. Frequent ascitic fluid drainage was required to control the symptom of abdominal distension.

Hemodialysis is a prevalent modality in cirrhotic patients with ESRD; however, difficulties of managing hemodynamic instability, coagulopathy, and malnutrition are common.^[9]^ Recently, PD has been reported to have the potential to overcome hemodialytic complications and manage large-volume ascites in cirrhotic patients, with comparable mortality, infection, and mechanical complication rates to the control PD population.^[3,7,8]^ Furthermore, cirrhotic patients undergoing PD have retrospectively shown lower all-cause mortality compared with those undergoing HD.^[10]^ Therefore, PD may become a feasible therapeutic option for cirrhotic patients with ESRD. However, PD is associated with a rare but fatal complication of EPS, bowel obstruction with peritoneal fibrosis. The mesothelial-mesenchymal transition of peritoneal mesothelial cells stimulated by proinflammatory cytokines is thought to be a key process leading to peritoneal fibrosis and the development of EPS,^[11]^ although clear mechanisms remain unclear. PD duration has a strong association with the risk of EPS, which almost always occurs in patients treated for more than 5 years.^[2,12]^ Frequent or prolonged peritonitis and higher peritoneal solute transport rate are also possible risk factors for EPS.^[3]^ While liver cirrhosis has also been reported to carry a risk of EPS. Although the pathogenesis of EPS under cirrhosis has been poorly understood, the underlying persistent intraabdominal infections due to spontaneous bacterial peritonitis might lead to peritoneal fibrosis and membrane formation, resulting in the development of EPS.^[4,5]^

Of note, there have been only a few reports demonstrating EPS complicated with both PD and cirrhosis.^[13]^ In the autopsy case, the patient who had a 1-year history of PD died 32 hours after admission with the findings of EPS and non-occlusive mesenteric infarction, while the clinical course of cirrhosis and ESRD until the development of EPS is not clearly described. In our case, intraperitoneal inflammation owing to repeated peritonitis is the possible process for peritoneal membrane fibrosis, resulting in the early development of EPS. Our patient successfully survived an operation. This and our reports indicate that concurrent cirrhosis and PD, both of which potentially have underlying conditions of intraperitoneal inflammation, are associated with a risk of developing EPS after a short period of PD.

The positive effect of immunosuppression including corticosteroid on EPS has been reported to target the inflammatory components of EPS.^[14]^ However, the patients with cirrhosis are prone to develop sepsis, sepsis-induced organ failure, and death,^[15,16]^ and there is no evidence evaluating the effect of immunosuppression on EPS in the cirrhotic population. In the present case, we did not use immunosuppressive drugs to avoid infections, but selected surgery which has favorable outcomes to decrease 5-year overall and EPS related mortality rates to 34% and 20%, respectively.^[17]^ The operation successfully rescued the patient, while we still need to beware of the postoperative recurrence, which is up to 23.9%.^[17]^ In addition, the progression of cirrhotic complications, including hemodynamic instability, coagulopathy, malnutrition, and ascites, will make future hemodialytic management more difficult. Further reports are needed for suitable management of EPS and underlying cirrhosis and ESRD. Nevertheless, it is worth keeping in mind that cirrhotic patients with ESRD can develop EPS after a short duration of PD, to avoid a fatal condition and prolong the window of opportunity for transplantation.

In conclusion, our report demonstrated that the cirrhotic patient with ESRD developed EPS after 4 years of PD. Surgical therapy successfully rescued the patient. Concurrent cirrhosis and PD could lead to EPS after a short period of PD.

## Acknowledgments

The authors thank Jane Charbonneau, DVM, from Edanz (https://jp.edanz.com/ac) for editing a draft of this manuscript.

## Author contributions

KW-K and YK managed and followed up the patients’ care. JG performed surgery and followed up the perioperative care. KW-K contributed to the conception and design of the work, and wrote and summarized the manuscript. YK, JG and KK revised the manuscript critically. All authors have read and approved the manuscript.

**Conceptualization:** Kanako Watanabe-Kusunoki.

**Writing – original draft:** Kanako Watanabe-Kusunoki.

**Writing – review & editing:** Yoshihiro Kusunoki, Junichi Goto, Kazutaka Kukita.
